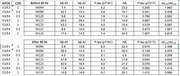# Change of plasma biomarkers after gamma frequency of white light intervention in Alzheimer's dementia

**DOI:** 10.1002/alz70856_098732

**Published:** 2025-12-24

**Authors:** Yuan‐Han Yang, Ling‐Chun Huang, Ching‐Fang Chien, Ping‐Song Chou

**Affiliations:** ^1^ Kaohsiung Medical University Gangshan Hospital, Kaohsiung, Taiwan; ^2^ Kaohsiung Medical University Hospital, Kaohsiung Medical University, Kaohsiung City, Taiwan; ^3^ Neuroscience Research Center, Kaohsiung Medical University, Kaohsiung, TAIWAN, Taiwan; ^4^ Kaohsiung Medical University Hospital, Kaohsiung, Taiwan; ^5^ Kaohsiung Medical University, Kaohsiung, Taiwan

## Abstract

**Background:**

Previous study using white light with gamma oscillations, 40 Hz, through our device, M+, has shown the benefit to slow the clinical decline and reduce caregiver burdens. Meanwhile, the cell‐line studies have revealed the gamma oscillation of light can reduce the phosphorylation of tau protein and the aggregation of beta‐amyloid 1‐42 (Aβ_1‐42_), and can enhance the function of glial cell to phagocytize the debris of beta‐amyloid (Aβ) to release the cerebral pathological burden of Alzheimer's disease (AD). Recently plasma biomarker such as ratio of Aβ_1‐40_ / Aβ_1‐42_, *p*‐tau_181_, and *p*‐tau _217_ have regarded as biomarkers can reflect the cerebral amyloid and tau pathological burden to reflect the clinical status. We are going to examine whether gamma oscillation of light can change the plasma biomarkers to apart from psychometrics for their clinical effects.

**Method:**

Seven clinically diagnosed AD patients have recruited from the outpatient department of an area hospital. The comprehensive neuropsychological examinations, mini‐mental status examination (MMSE), cognitive ability screen instrument (CASI), clinical dementia rating (CDR), neuropsychiatric inventory (NPI), were administered at the baseline and the 6th months together with the blood sample obtained for plasma biomarkers detection. The plasma Aβ_1‐40_, Aβ_1‐42_, *p*‐tau_181_, and *p*‐tau _217_ were examined by ELISA. Desk lamp, M+, was used at each patient's home with at least 1 hour per day till 6 months

**Result:**

The 7 clinically diagnosed AD patients, 4 with clinical dementia rating (CDR) 1 and 3 with CDR0.5, with their mean age 80.8 years old receiving the white light with gamma oscillation as desk lamp have recruited. For the very limited sample size, there is no obvious improvement of psychometrics have founded but for plasma biomarkers: *p*‐tau_181_, 5 out of 7 individuals did not increase its plasma level as well as did in 6 out of 7 in *p*‐tau _217_ level after the light intervention.

**Conclusion:**

Our pilot study has provided the objective evidences that 40 Hz light intervention through our M+ device can modify the plasma biomarkers for AD. Owing to limited sample size, a large‐scale randomized case‐control study is necessary to examine these effects